# Cytotoxic Activities of *Physalis minima* L. Chloroform Extract on Human Lung Adenocarcinoma NCI-H23 Cell Lines by Induction of Apoptosis

**DOI:** 10.1093/ecam/nep057

**Published:** 2011-01-11

**Authors:** Ooi Kheng Leong, Tengku Sifzizul Tengku Muhammad, Shaida Fariza Sulaiman

**Affiliations:** ^1^School of Biological Sciences, Universiti Sains Malaysia, 11800 Minden, Penang, Malaysia; ^2^Malaysian Institute of Pharmaceuticals and Nutraceuticals, Ministry of Science, Technology and Innovation, SAINS 10, Persiaran Bukit Jambul, 11900 Penang, Malaysia; ^3^Department of Biological Sciences, Faculty of Science and Technology, Universiti Malaysia Terengganu, 21030 Kuala Terengganu, Terengganu, Malaysia

## Abstract

*Physalis minima* L. is reputed for having anticancer property. In this study, the chloroform extract of this plant exhibited remarkable cytotoxic activities on NCI-H23 (human lung adenocarcinoma) cell line at dose- and time-dependent manners (after 24, 48 and 72 h of incubation). Analysis of cell-death mechanism demonstrated that the extract exerted apoptotic programed cell death in NCI-H23 cells with typical DNA fragmentation, which is a biochemical hallmark of apoptosis. Morphological observation using transmission electron microscope (TEM) also displayed apoptotic characteristics in the treated cells, including clumping and margination of chromatins, followed by convolution of the nuclear and budding of the cells to produce membrane-bound apoptotic bodies. Different stages of apoptotic programed cell death as well as phosphatidylserine externalization were confirmed using annexin V and propidium iodide staining. Furthermore, acute exposure to the extract produced a significant regulation of c-myc, caspase-3 and p53 mRNA expression in this cell line. Due to its apoptotic effect on NCI-H23 cells, it is strongly suggested that the extract could be further developed as an anticancer drug.

## 1. Introduction

Lung cancer remains a major global health problem, accounting for more than a million annual deaths worldwide [1]. It is twice the death rate of the second-most prevalent cancer, that is, prostate cancer in men [2]. The incidence of lung cancer can be correlated with the age of both males and females and there is still lack of effective drugs to treat this disease [3].

Herbal formulation consisting of single and multiple of herbs is commonly prescribed as an alternative way to treat cancer. An anticancer plant that was selected for this study is *Physalis minima* L. The decoction of the whole plant is taken orally to treat cancer and the leaves are used as a poultice for ulcer [4, 5]. This herb is commonly known as the bladder cherry (Leletup-direct translation from Malay) and belongs to the Solanaceae family [5]. Its reputed efficacy in treating cancer has been validated (*in vitro*) against CORL23 lung and MCF7 breast cancer cell lines, but the mechanisms underlying the anticancer activity still remain unknown. Two active anticancer compounds have been isolated from its methanol extract of stem and leaf and were identified as physalin B and physalin F [6]. However, these two compounds were found to exhibit non-selective cytoxicity against all tested human cell lines including non-cancerous cell with physalin F consistently more active [6, 7].

Recently, intensive studies have been conducted to examine the mechanism responsible for the anticancer effects of plant-based drugs. Apoptosis is a specific mode of cell death that can only target cancer cell with little or no damage to non-cancerous cells [8]. It helps in reducing the incidence of side effect in patients [9]. Information on the apoptotic effect elicited by the extracts and bioactive compounds of *Physalis* sp. are still limited to a few findings, such as the cell death signaling effects of physalins B and F on PANC-1 pancreatic cancer cells. They were reported as potent inhibitors for the aberrant hedgehog (Hh)/GLI signaling pathway (that causes formation and progression of various cancers) by inhibiting GL2-mediated transcriptional activation, decreasing hedgehog-related component expression and reducing the level of anti-apoptotic Bcl-2 gene expression [10]. Moreover, apoptotic induction in human lung cancer H661 cells by the supercritical carbon dioxide extract of *Physalis peruviana* was associated with cell cycle arrest at the S phase, mediated through the p53-dependent pathway and modification of pro-apoptotic protein (Bax) and inhibitor of apoptosis protein (IAP) expression [11]. In addition, the ethanol extract of *P. peruviana* was found to induce apoptosis on human liver cancer Hep G2 cells through CD95/CD95L system and the mitochondrial signaling transduction pathway [12]. Furthermore, the methanol extract of *Physalis angulata* induced apoptosis and arrested human breast cancer MAD-MB 231 cells at G2/M phase [13] and induced apoptosis in human oral cancer HSC-3 cells through oxidative stress-dependent induction of protein expression such as heme oxygenase-1 and Cu/Zn superoxide dismutase [14].

Based on our previous comparative cytotoxicity studies of the extracts and fractions (obtained from the chloroform extract) of *Physalis minima*, it has been established that the anti-proliferative activity of the chloroform extract in human lung adenocarcinoma NCI-H23 cells was relatively better than other extracts and its fractions [15]. Therefore, it was selected for this study with aims (i) to evaluate the effect of the extract to NCI-H23 cells proliferation in different incubation periods and (ii) to determine the cell death mechanism elicited by the extract *via* morphological and molecular investigations.

## 2. Methods

### 2.1. Chemicals

The DeadEnd Colometric Apoptosis Detection System was purchased from Promega, USA. The Annexin-V-FLOUS kit was purchased from Roche Diagnostics, Germany. The methylene blue assay, dimethyl sulfoxide (DMSO) and propidium iodide were obtained from Sigma Aldrich, USA. All culture media and additives were from Hyclone, USA. All other chemicals were reagents of molecular grade, as appropriate.

### 2.2. Preparation of Crude Extracts

The *P. minima* plant was collected from Arau-Perlis, Malaysia. The plant was identified and verified by Mr V. Shunmugam of Universiti Sains Malaysia. The voucher specimen (no. 11001) was preserved and deposited in the herbarium of School of Biological Sciences, Universiti Sains Malaysia.

The whole plant materials were washed, dried and chopped finely using a grinder. The dried material was then transferred into the Soxhlet extractor. The dried plant material was exhaustively extracted with chloroform by Soxhlet extraction. The extracts were filtered and concentrated using rotary evaporator, and then evaporated to dryness. The dried extracts were then weighed using microbalances (Sartorius, Germany) and reconstituted with 99.9% (v/v) DMSO to prepare a stock solution at a concentration of 10 mg/mL. The stock solution was serially diluted to eight different working concentrations. As for the positive control, the stock solution of vincristine sulfate (a commercial drug) at a concentration of 1 mg/mL was prepared using DMSO and diluted serially to 24 different concentrations.

### 2.3. Cell Line and Culture Medium

NCI-H23 (human lung adenocarcinoma) cell line was obtained from American Type Cell Culture (ATCC), USA, and cultured in RPMI 1640, supplemented with 2 mM l-glutamine, 10% (v/v) fetal calf serum (FCS), 100 U/mL penicillin and 100 *μ*g/mL streptomycin, as recommended by ATCC.

### 2.4. *In Vitro* Cytotoxicity Assay

Nearly confluent cultures of cells were harvested with 0.05% (w/v) Trypsin-EDTA. Cells were then centrifuged and pellet resuspended with a complete medium with 10% (v/v) FCS. Then, 100 *μ*L of cells were plated into each well of 96-well plate at a density of *∼*6000 cells/well. Cell viability was routinely determined using the trypan blue exclusion test to ensure it was always in excess of 95%. The cells were then allowed to attach and incubate at 37°C in a CO_2_ incubator for a further 24–48 h. After the cells reached 80–90% confluence, the medium was removed and replaced with medium containing only 0.5% (v/v) FCS and the cells were incubated for further 4 h. The reason for this was for the cells to achieve quiescent. Subsequently, the cells were treated with different concentrations of extracts. Controlled cells were cultured in a 0.5% (v/v) FCS-containing medium alone. Of serial diluted extracts, 1 *μ*L was added into each well. After treatment, the plates were incubated for 72 h. Vincristine sulfate was used as a positive control.

Cell survival was determined by the procedure using methylene blue staining as described by Yamazaki et al. [16] and Li and Hwang [17]. After 72 h of incubation with plant extracts, the surviving cells were fixed with 2.5% (v/v) glutaraldehyde for 15 min and were then washed with a 0.15 M sodium chloride (NaCl) solution to remove the dead cells. The fixer cells subsequently were stained with 100 *μ*L of 0.05% (w/v) methylene blue solution for 15 min. After washing off the excess dye with NaCl, dye elution was carried out using 200 *μ*L of 0.33 M hydrochloric acid (HCl). Absorbance was read at 650 nm using *V*_max_ Kinetic Microplate Reader (Molecular Devices, USA). The number of surviving cells was determined from the absorbance value.

### 2.5. Detection of DNA Fragmentation

Nuclear DNA fragmentation was detected using DeadEnd Colometric Apoptosis Detection System (Promega, USA). Briefly, cell suspensions were subcultured on Labtek Chamber Slides, and then incubated for 24–48 h. When the cells achieved confluency between 80% and 90%, the medium was replaced with a medium containing only 0.5% (v/v) FCS. The cells were then incubated at 37° C for 4 h and treated with the *P. minima* chloroform extract at a concentration of EC_50_ at 72 h (2.80 *μ*g/mL). Negative control cells were treated with the same concentration of DMSO. Positive control cells were treated with DNase I (1 U/mL) and vincristine sulfate at a concentration of EC_50_ at 72 h (0.0015 *μ*g/mL). In all cases, the final concentration of DMSO in each control slide did not exceed 1% (v/v). The slides were then incubated for 24 h. After treatment, the cells were rinsed twice with a phosphate-buffered saline (PBS), and then processed according to the DeadEnd Colometric Apoptosis Detection System protocol as described by the manufacturer (Promega, USA). Subsequently, the slides were observed using the light microscope (Olympus BH2 light microscope) and the images taken using the AnalySIS Docu SoftImaging Software, Germany.

### 2.6. Detection of Plasma Membrane Integrity

In order to detect the loss of plasma membrane of necrotic cells, the NCI-H23 cells were cultured on 6-well plates, and then incubated for 24–48 h. When the cells reached between 80% and 90% confluency, the medium was replaced with a fresh medium containing only 0.5% (v/v) FCS. The cells were then incubated for a further 4 h and treated with the *P. minima* chloroform extract at a concentration of EC_50_ at 72 h (2.80 *μ*g/mL). Negative control cells were treated with the same concentration of DMSO. The cells were rinsed with PBS and stained using the trypan blue exclusion assay after 24 and 72 h incubation period. Subsequently, the samples were viewed using an inverted phase contrast light microscope.

### 2.7. Ultrastructural Analysis

NCI-H23 cells were incubated with the *P. minima* chloroform extract at a concentration of EC_50_ at 72 h (2.80 *μ*g/mL) for 24 h. Negative control cells were treated with the similar concentration of DMSO. After incubation, the cells were trypsinized, centrifuged and then resuspended twice with PBS. The cells were then rinsed, pelleted and then resuspended in the McDowell-Trump fixative solution [containing 4% (v/v) formaldehyde and 1% (v/v) glutaraldehyde in a 0.1 M phosphate buffer, pH 7.3] at 4°C for 24 h. The cells were pelleted and rinsed in a 0.1 M phosphate buffer for 10 min and repeated three times. Post-fixed was carried out with 1% (w/v) osmium tetroxide prepared in a 0.1 M phosphate buffer for 1-2 h at room temperature. The cells were then dehydrated with 50% (v/v) ethanol for 15 min, followed by 75% (v/v) ethanol for 15 min, 95% (v/v) ethanol for 15 min and repeated, 100% (v/v) ethanol for 30 min and repeated and, lastly, 100% (v/v) acetone for 10 min and repeated. Infiltration was conducted using the mixture of acetone : Spurr's resin mix (1 : 1) in a rotator for another 2-3 days with a daily change of Spurr's mix of each sample specimen. Subsequently, the cells were embedded and cured at 60°C for 12–48 h.

Sectioning was preceded by the analysis of a semi-thin section (1 *μ*m), stained at 40°C with 1% (w/v) toluidine blue. This was then followed by sectioning of thin sections (<1 *μ*m), collected using copper grids. Reichart Supernova Ultra Microtome was used to produce the sections. The thin sections were stained with uranyl acetate for 15 min by complete immersion, in a covered Petri-dish lined with dental wax. Lastly, the sections were stained with lead citrate using the same procedures. The samples were examined and observed under a Philips CM 12 transmission electron microscope (TEM).

### 2.8. Detection of Phosphatidylserine Externalization

NCI-H23 cells were incubated with the *P. minima* chloroform extract at a concentration of EC_50_ at 72 h (2.80 *μ*g/mL) for 24 h. Negative control cells were treated with the similar concentration of DMSO. After treatment, the cell in the chamber slides were washed with PBS twice and subsequently processed according to the Annexin-V-FLUOS (Roche Diagnostics, Germany) (combination of annexin V and propidium iodide) protocol as described by the manufacturer's instruction. Subsequently, the slides were immediately analyzed using a fluorescence microscope with an excitation wavelength in the range of 450–500 nm and detection in the range of 515–565 nm (green). Photos were taken using the Olympus BH2-RFCA Fluorescence Microscope with Olympus camera attachment. Green fluorescence was observed in annexin V positive cells and red fluorescence in cells which uptake the propidium iodide dye.

### 2.9. Determination of Apoptotic-Related Genes Expression

In order to investigate the mRNA expression levels of c-myc, caspase-3 and p53, which are responsible to trigger apoptosis mechanism, semi-quantitative reverse transcription polymerase chain reaction (RT-PCR) was carried out. NCI-H23 cells were cultured in T25 flasks and starved with a medium containing only 0.5% (v/v) FCS for 4 h. The cells were then treated with the *P. minima* chloroform extract at a concentration of EC_50_ at 72 h (2.80 *μ*g/mL) for the period of 24 h. After treatment, the total cellular RNA was isolated using the Tri-Reagent LS (Molecular Research Center, USA) according to the manufacturer's instructions. Of the isolated total cellular RNA sample, 1 *μ*g was treated with DNase I, followed by reverse transcribed into cDNA and subjected to PCR amplification. The optimized PCR amplification program comprised an initial denaturation step at 94°C for 2 min, followed by denaturation at 94°C for 45 s, annealing at 56°C for 1 min, extension at 72°C for 2 min and final extension step at 72°C for 10 min. The PCR optimization process including the optimal cDNA concentration and the number of amplification cycles used to amplify c-myc, caspase-3, p53 and *β*-actin genes was in the exponential phase of PCR amplification (i.e. provide a linear relationship between the amount of amplification and the concentration of the original cDNA temples) (data not shown), indicating that the conditions were optimized for semi-quantitative studies [14, 15]. The sequences of primers for analysis were synthesized based on the human mRNA encoding the respective genes. The amplification products were separated on 1.2% (w/v) agarose gel and stained with ethidium bromide. Gene expressions signaling at each point of time were quantified using GeneTools analysis software on GENE GENIUS gel documentation system (Syngene, UK). The signals of c-myc, p53 and caspase-3 were normalized fromc *β*-actin and the ratio in unstimulated samples was assigned as 1. The housekeeping gene *β*-actin was used as an internal standard control.

### 2.10. Calculation and Statistics

Cytotoxicity experiments were performed in six replicates and results were expressed as percentage growth inhibition of control. EC_50_ values for growth inhibition was derived from a nonlinear model (curvfit) based on the sigmoidal dose response curve (variable) and computed using GraphPadPrism, USA. Data are given as mean ± SEM. The criterion of cytotoxic or non-cytotoxic was adapted from the guidelines set by the National Cancer Institute (NCI) which indicated that plant extracts with EC_50_ ≤ 20 *μ*g/mL were considered to be cytotoxic and non-cytotoxic if otherwise [18].

DNA fragmentation experiments were conducted in triplicate. The cells from five random microscopic fields were counted to get the percentage of TUNEL positive cells (apoptotic index, AI% = number of apoptotic nuclei/number of nuclei scored × 100%) using 200x magnifications and the mean percentage ± SEM was calculated. The significant differences between the mean percentage of positive stained nuclei of control cells and treated cells were determined using one-way ANOVA, computed using GraphPadPrism (Graphpad, USA).

The significant differences in the ratio to *β*-actin (between each time point and control) were determined using one-way ANOVA and Dunnett's Multiple Comparison Test for post-comparison tests, computed using GraphPadPrism software (GraphPad, USA). Differences were considered to be significant if *P* < .05.

## 3. Results

### 3.1. Cytotoxic Effect of the Chloroform Extract of *P. minima* on NCI-H23 Cells

**Figure 1:**
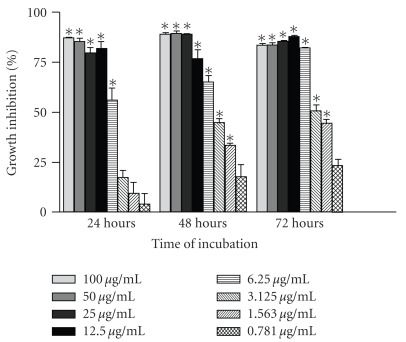
Effect of *P. minima* chloroform extract on proliferation of NCI-H23 cells at 24, 48 and 72 h. Each value represented mean ± SEM of six replicates (*n* = 6); *P < .05.

Dose-dependent inhibitions in NCI-H23 cells were observed when the cells were treated with this extract at all incubation intervals (Figure 1). Interestingly, the chloroform extract produced >75% of growth inhibition in NCI-H23 cells, which appeared to be relatively unchanged at higher concentrations (25–100 *μ*g/mL) for all time points (Figure 1). After 24 h of treatment, only at a concentration of 6.25 *μ*g/mL and above, the chloroform extract significantly inhibited the growth of NCI-H23 cells (*P* < .05). Meanwhile, after 48 and 72 h of incubation, at most tested concentrations (100–1.563 *μ*g/mL), the extract exhibited significant inhibitory effects (*P* < .05). The inhibitory activities increased and the EC_50_ values decreased as the incubation periods were increased from 24 h (5.28 *μ*/mL) to 48 h (3.55 *μ*g/mL) and 72 h (2.80 *μ*g/mL). However, the EC_50_ for vincristine sulfate (0.0015 *μ*g/mL) was very much lower as compared to the EC_50_ deduced from the chloroform extract at 72 h incubation period (data not shown).

### 3.2. Effect of *P. minima* Chloroform Extract on Cell Death of NCI-H23 Cells

**Figure 2:**
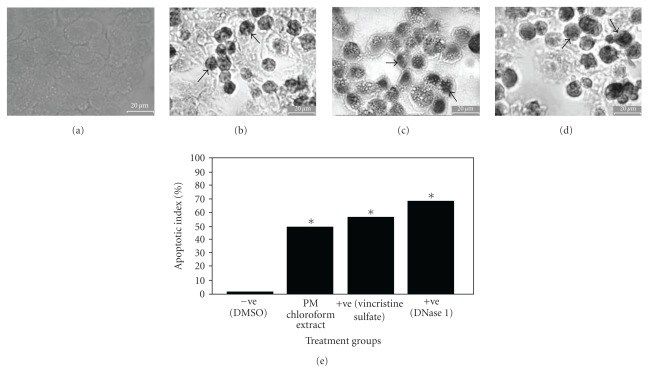
The effect of (a) DMSO l% (v/v), (b) *Physalis minima* chloroform extract (2.80 *μ*g/mL, EC_50_ a 72 h), (c) DNase I (1 U/mL) and (d) vincrstine sulphate (0.0015 *μ*g/mL, EC_50_ at 72 h) or NCI-H2: cells for 24 h and subjected to Deadend Colometric Apoptosis Detection System (Promega, USA) Apoptotic cells with stained nuclei were rnarked by arrows. (e) Comparison of the mean percentage of apoptotic index between DNase 1-, vincristine sulphate- and *P. minima* chloroform extract-treated cells to untreated cells (DMSO) at 24 hours treatment in different cell lines. Each value represented mean ± SEM from three independent experiments. *P < .05 (as compared with negative control).

**Figure 3:**
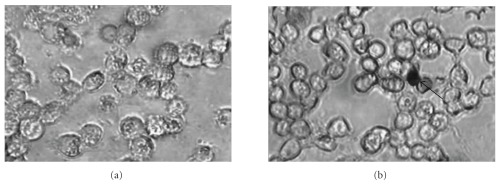
Trypan blue staining of NCI-H23 cells treated with *P. minima* chloroform extract (2.80 *μ*g/mL, EC_50_ at 72 h) post 24 (a) and 72 h (b) (Original magnification × 200). Necrotic cells were marked by arrow.

In order to investigate whether apoptosis may play an important role in mediating the cell death of NCl-H23 cells elicited by the chloroform extract of *P. minima*, the fragmented genomic DNA was detected using a modified TUNEL assay. As shown in Figures 2(b), 2(c) and 2(d), the extract-treated NCl-H23 cells produced dark brown stained nuclei with similar observation found in the positive control cells treated with DNase I and vincristine sulfate. The nuclei were specifically stained and evenly distributed. Most of the positive stained nuclei were rounded or oblong in shape. But almost all nuclei of untreated negative control cells were not stained with this assay (Figure 2(a)). The mean percentage of apoptotic index for the extract-treated NCl-H23 cells was 49.89% and significantly different as compared to the negative control (DMSO) (1.39%) (*P* < .05) (Figure 2(e). Comparatively, the percentage was lower than that in positive controls (68.41% and 56.40% for DNase I and vincristine sulfate, resp.). This result strongly indicated that apoptosis was one of the possible type of cell death in NCl-H23 cells after 24 h exposure to the chloroform extract. The trypan blue exclusion assay showed that only a few NCl-H23 cells were positively stained after treated with the extract for 24 and 72 h. The results strongly suggested that the surface of most treated cells was intact after incubation with the extract at both time points (Figures 3(a) and 3(b). Therefore, the mode of cell death elicited by the chloroform extract of *P. minima* in NCI-H23 cells was unlikely via necrotic mechanism in nature.

**Figure 4:**
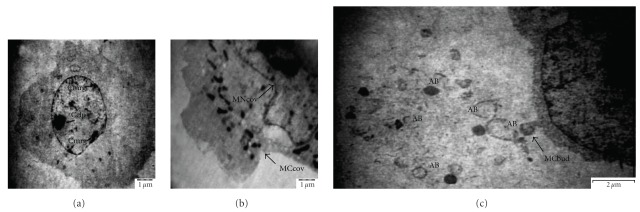
Morphological features of apoptosis in NCI-H23 cells after treated with *Physalis minima* chloroform extract (2.80 *μ*g/mL, EC_50_ at 72 h) for 24 h. (a) Chromatin clumping (Cclp), chromatin margination (Cmrg), (b) convolution of nuclear membrane (MNcov) and cytoplasmic outlines (MCcov), (c) budding of cell (MCbud) and led to the formation of apoptotic bodies (AB), were observed (shown by arrows).

TEM analysis exhibited different morphological alterations in NCI-H23 cells after treatment with the chloroform extract of *P. minima* for 24 h. Characteristics of apoptotic cell death were observed in the majority of treated NCI-H23 cells. There was clumping of the nuclear chromatin into more densely packed material that becomes marginated against the nuclear membrane (Figure 4(a)). This was accompanied by a convolution of nuclear envelope and cytoplasmic membrane (Figure 4(b)). However, the cytoplasmic membrane and cell organelles remained intact. Budding of cells was clearly demonstrated (Figure 4(c)), where the cytoplasmic membrane formed extensions and separated. The plasma membrane was then sealed to produce membrane-bound apoptotic bodies that contained cellular material of the cells in various combinations. These ultrastructural analysis results clearly indicated that apoptosis appeared to be the dominant mechanism of cell death exerted by the chloroform extract of *P. minima* in NCI-H23 cells. As for negative control, the DMSO-treated cells did not show these morphological characteristics (data not shown).

**Figure 5:**
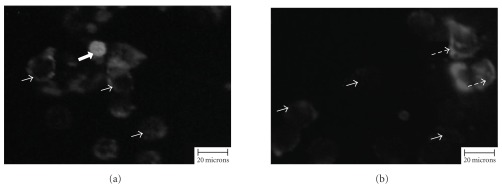
The effect of *P. minima* chloroform extract (2.80 *μ*g/mL at 72 h) on NCI-H23 cells for 24 h and subjected to Annex-V-FLUOS kit (Roche, Germany). A weakly scattered annex in V staining pattern of cell surface was evident in most of the cells (thin arrows). Only a few of homogenous-high intensity of annex in V fluorescence cells (thick arrow) and both annex in V-propidium iodide stained cells (green and red fluorescence) (dotted arrows) were also observed.

The existence of apoptotic programed cell death elicited by chloroform extract of *P. minima* in NCI-H23 cells was confirmed using Annexin-V-FLUOS assay (Roche, Germany). The early phase of apoptosis or programed cell death was evident in most of the cells as shown in Figures 5(a) and 5(b), with weakly scattered fluorescent annexin V (green). Only a few homogeneous and high intensity annexin V staining pattern was observed, indicating a later phase of apoptosis or programed cell death (Figure 5(a)). Meanwhile, some cells were positively stained with both annexin V and propidium iodide, which referred to as late stage apoptosis (secondary necrosis) and programed cell death (Figure 5(b)). However, the DMSO-treated cells (negative control) did not exhibit these staining patterns (data not shown). Thus, the different stages of apoptosis or programed cell death were evident in NCI-H23 cells treated with the chloroform extract of *P. minima*.

### 3.3. Effect of *P. minima* Chloroform Extract on Apoptosis-Related Genes Expression Level of NCI-H23 Cells

**Figure 6:**
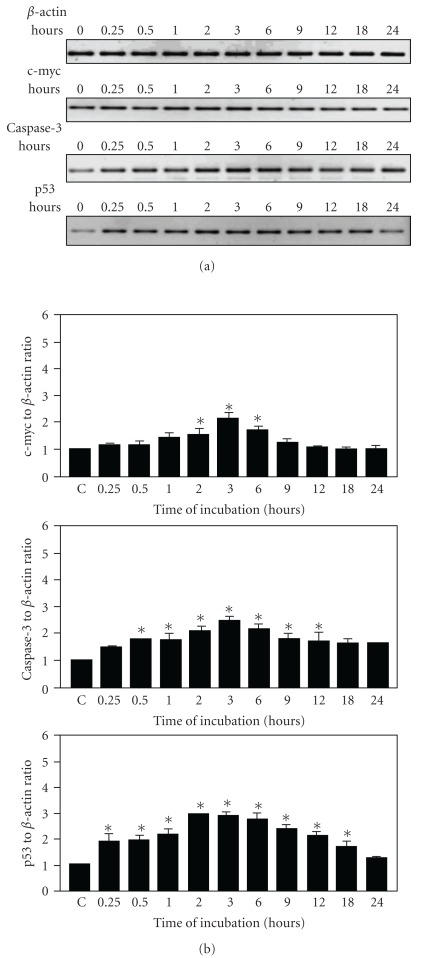
(a) Time course mRNA expression of *β*-actin, c-myc, caspase-3 and p53 in NCI-H23 cells incubated in the absence or presence of *P. minima* chloroform extract. *β*-actin was used as an internal standard control for each PCR reation. (b) Semi-quantitative analysis of the c-myc, caspase-3 and p53 mRNA level in NCI-H23 treated with *P. minima* chloroform extract using densitometric scanning. Each value represented mean ± SEM (*n* = 3 at each point) of the ratio of RT-PCR product of the respective genes to *β*-actin, assigning the ratio in unstimulated cells as 1. *P < .05 versus C (control).

Semi-quantitative RT-PCR was used in this study in order to determine the expression levels of apoptosis-related genes in *P. minima* chloroform extract-treated NCl-H23 cells. As shown in Figures 6(a) and 6(b), the expression of c-myc at rest (0 h) was not much different from stimulated cells at 15 min and 30 min and slightly increased for the next 90 min before reaching a peak at 3 h (2.1-fold increase). However, the expression of c-myc was then reduced at 6 h (1.7-fold increase) and gradually decreased to baseline levels at 18 and 24 h. The mRNA expression of c-myc was statistically significant at 2, 3 and 6 h post-stimulation as compared to untreated cells (*P* < .05) (Figure 6(b)).

As for the caspase-3 gene, the mRNA expression was induced as early as 15 min and still increased at 30 min but declined minimally for the next 30 min and gradually reaching its peak at 3 h with an ∼2.4-fold increase (Figures 6(a) and 6(b)). However, the mRNA expression of caspase-3 was slightly reduced for the next 3 h (2.0-fold increase) and decreased steadily to the lowest levels at 18 and 24 h time points. When compared to control cells, the marked increase of caspase-3 mRNA expression was proven to be statistically significant from 30 min to 12 h post-stimulation (*P* < .05) (Figure 6(b)).

By contrast, the level of p53 mRNA expression was very low at rest (0 h) but increased dramatically at 15 min and remained elevated for the next 75 min before reaching the highest peak at 2 h (2.8-fold increase) (Figures 6(a) and 6(b)). The high mRNA expression of p53 appeared to sustain until the ninth hour and declined slightly at 12 h and eventually decreased to the lowest level at 24 h. In addition, the p53 expression was statistically higher than that control cells from 15 min to 18 h (*P* < .05) (Figure 6(b)).

Overall, NCl-H23 cells treated with the chloroform extract of *P. minima* exhibited high mRNA expression levels of c-myc, caspase-3 and p53 within 2–6 h post-stimulation. Caspase-3 and p53 genes seemed to have similar expression patterns, whereby their expression was observed at early post-stimulation. Moreover, the induction of p53 and caspase-3 mRNA expression was slightly higher as compared to that of c-myc gene. Thus, there was a clear indication that the cell death induced by the chloroform extract of *P. minima* in NCl-H23 cells was mediated via the activation of c-myc, caspase-3 and p53 gene expression.

## 4. Discussion

It has previously been shown that treatment using *P. minima* extracts and compounds inhibited cell proliferation, but the mechanism of cell death remained unclear [6]. In this study, we found that the chloroform extract was able to inhibit cell proliferation and induce apoptosis. These were confirmed by six independent methods, namely the methylene blue assay for cytotoxicity evaluation, DeadEnd Colometric System (Promega, USA) to label the apoptotic nuclei cells, trypan blue exclusion assay to detect the loss of plasma membrane integrity of necrotic cell, TEM analysis to describe the ultrastructural or micro morphology characteristics of the apoptotic cell, annexin V and propidium iodide staining to detect the stages of apoptosis and RT-PCR analysis to determine the mRNA expression level of apoptotic gene.

The extract was found to significantly abrogate the growth of NCI-H23 cells in a dose- and time-dependent manner. Based on our chromatographic and spectroscopic analyses, the extract was found to contain physalins B, F and K [15]. These isolated physalins which are commonly found in genus *Physalis* were reported with a cytotoxic effect against numerous human cell lines [7, 19, 20]. The increase in activity of the chloroform extract as compared with their fractions might be due to the synergistic effect of the various physalins in the extract. The occurrence of apoptosis was initially detected based on the percentage of apoptotic index of the extract-treated NCI-H23 cells that was significantly higher than that in untreated cells. DNA fragmentation exhibited in TUNEL assay-labeled nuclei cells is commonly used as a biochemical index of apoptosis [21]. Analysis of plasma membrane permeability using the trypan blue exclusion assay has ruled out necrosis as the cause of cell death in extract-treated NCI-H23 cells. The cross-sections of apoptotic cells were also observed using TEM analysis of the extract-treated NCI-H23 cells. It was recognized by stereotypical morphological changes such as shrinking and deformation of cells, blebbing of cytoplasmic membrane, clumping, condensation and margination of nuclear chromatin, followed by breaking up of the cells into small membrane-enclosed apoptotic bodies [22]. The observation of apoptotic-morphological features was clearly established in various studies involving anticancer agents such as cisplatin and flavopiridol that induced apoptosis in C6 glioma cells [23] and A172 glioma cells, respectively [24].

The apoptotic programed cell death elicited by the chloroform extract was also confirmed using the Annexin-V-FLUOS assay (Roche, Germany). Annexin V is a Ca^2+^-dependent phospholipid-binding protein that detects the phosphatidylserine externalization of the plasma membrane [25]. With fluorescence microscopy observation, different annexin V staining patterns have been observed that indicated different phases of apoptosis in the extract-treated cells. The faintly annexin V-stained cells probably due to a limited phosphatidylserine exposure during the early stage of apoptosis [26, 27]. The heavily stained annexin V and propidium iodide cells indicated the later phase of the apoptotic process, whereby the cells lost their plasma membrane integrity and more binding sites of phosphatidylserine were detected [28].

The roles of c-myc, caspase-3 and p53 mRNA levels in the chloroform extract-induced apoptotic pathways were further verified based on their expression profiles at different times of treatment. Both p53 and caspase-3 were significantly induced by the chloroform extract at the earlier phase of treatment (15 min and 30 min, resp.). Their expressions were sustained until the later stages of treatment and the results provide strong evidences to support the roles of these genes and their crosstalk in mediating the apoptosis-inducing effects of the extract. It has been well established that p53- and caspase-mediated apoptosis of most cells are induced through the activation of the death receptor (extrinsic) or the mitochondrial (intrinsic) pathway [29]. Overexpression of p53 was reported to provoke the extrinsic apoptotic pathway *via* the induction of death receptor proteins such as Fas and TRAIL, which transmitted the apoptotic death signal from the cell surface to intracellular signaling pathways [30, 31]. The pro-apoptotic function of p53 was also considered to be associated with the mitochondrial pathway that generally involves an induction of mitochondrial permeability transition and the subsequent release of apoptogenic factors such as cytochrome *c* that leads to caspase-9 activation, which in turn cleaves and activates effector caspases such as caspase-3 [32].

p53 was found to be induced 15 min earlier than caspase-3. This finding suggests that p53 activation may be an upstream cellular event that leads to mitochondrial membrane potential disappearance and caspase-3 may be responsible in executing the downstream process of apoptosis that mainly contributed to the typical morphological changes and DNA fragmentation in apoptotic cells [33]. The activation of caspase-3 has been reported in p53-induced apoptosis in various systems [34, 35]. Caspase-3-activated DNase that was induced by Fas-ligand or c-myc was discovered to be associated with caspase-3 during apoptosis [36]. The activated DNase was allowed to enter the nucleus and caused oligonucleosomal DNA fragmentation [37]. In agreement with this study, the ethanolic extract of *P. peruviana* was reported to induce Hep G2 cells apoptosis through the activation of caspase-3 while the elevation of p53 expression and caspase-3 activation were observed in its supercritical carbon dioxide extract-treated H661 cells [11, 12].

Meanwhile, c-myc gene only demonstrated a transient mRNA expression (2.1-fold increase) in extract-treated NCI-H23 cells at 3 h of treatment. The transient increment of this gene might enhance the apoptotic effect of p53 and caspase-3 with marked peak expression of these genes at 2-3 h of treatment. This is supported by a finding that suggested c-myc gene as a strong inducer of cell death *via* induction of several pro-apoptotic signal transduction mechanisms [38]. This gene has been described as a repressor to reduce ubiquitination of p53 that leads to the accumulation of p53 and subsequently triggers caspase-3 [31]. c-myc was reported to induce apoptosis by destabilizing mitochondrial integrity [39]. The upregulation of c-myc was essential to enhance the pro-apoptotic proteins of Bcl-2 family and suppresses their anti-apoptotic effects [38, 39].

**Figure 7:**
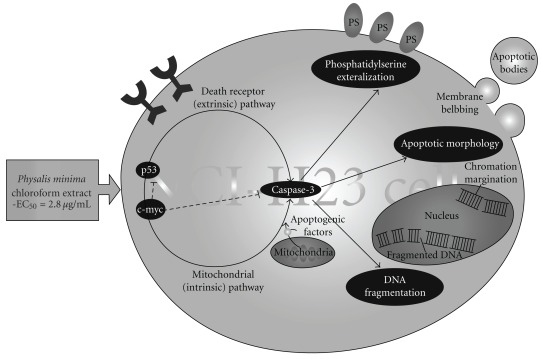
An Overview of apoptotic effects elicited by *P. minima* chloroform extract on NCI-H23 cells. The apoptotic mechanism was mediated by the activation of p53 (inducer and regulator), caspase-3 (executioner) and c-myc (enhancer dotted lines) which were reputed as main molecular regulators in both extrinsic and intrinsic pathway. This was subsequently resulting in biochemical and morphological alterations, including DNA fragmentation; phosphatidylserine (PS) externalization, chromatin migration, membrane blebbing and apoptotic bodies.

In conclusion, this study could offer scientific basis for the further in-depth evaluation of the chloroform extract of *P*. *minima*. It inhibited the proliferation of NCI-H23 cells at dose- and time-dependent manner *via* an apoptotic programed cell death. The induction of apoptotic cell death was suggested to be mediated *via* p53-, caspase-3- and c-myc-dependent cell apoptotic pathways. Based on the results obtained in this study, we constructed a mode of action as depicted in Figure 7. The model proposes that the chloroform extract induced the expression of p53 that resulting in the release of apoptogenic factors that would facilitate the cell apoptosis through the activation of caspase cascade.
